# How effective is good domestic kitchen hygiene at reducing diarrhoeal disease in developed countries? A systematic review and reanalysis of the UK IID study

**DOI:** 10.1186/1471-2458-8-71

**Published:** 2008-02-22

**Authors:** Anna Stenberg, Clare Macdonald, Paul R Hunter

**Affiliations:** 1 School of Medicine, Health Policy and Practice, University of East Anglia, Norwich NR4 7TJ, UK

## Abstract

**Background:**

To assess whether domestic kitchen hygiene is an important contributor to the development of diarrhoea in the developed world.

**Methods:**

Electronic searches were carried out in October 2006 in EMBASE, MEDLINE, Web of Knowledge, Cochrane central register of clinical trials and CINAHL. All publications, irrespective of study design, assessing food hygiene practices with an outcome measure of diarrhoea were included in the review. All included studies underwent data extraction and the data was subsequently analysed. The analysis was conducted by qualitative synthesis of the results. Given the substantial heterogeneity in study design and outcome measures meta-analysis was not done. In addition the existing dataset of the UK IID study was reanalysed to investigate possible associations between self-reported diarrhoea and variables indicative of poor domestic kitchen hygiene

**Results:**

Some 14 studies were finally included in subsequent analyses. Of the 14 studies included in this systematic review, 11 were case-control studies, 2 cross-sectional surveys, and 1 RCT. Very few studies identified any significant association with good environmental kitchen hygiene. Although some of the variables in the reanalysis of the UK IID study were statistically significant no obvious trend was seen.

**Conclusion:**

The balance of the available evidence does not support the hypothesis that poor domestic kitchen hygiene practices are important risk factors for diarrhoeal disease in developed countries.

## Background

Globally, diarrhoeal disease is estimated to affect some 4.5 billion people annually of whom an estimated 1.8 million will die, the large majority of these being children under the age of 4 years [1]. Although primarily a problem of developing countries diarrhoeal disease is also still a common problem in developed countries [2,3]. Although in developing countries the major cause of diarrhoeal disease is though to be due to contaminated drinking water in the developed world the main cause is either person to person transmission or foodborne [4,5].

Much public health legislation and activity has been directed at improving food hygiene in order to reduce the burden of foodborne disease in developed nations. However, one area where it has been difficult to legislate for is food hygiene practices in the home. Nevertheless direct advertising of home hygiene products has been strong for at least the last 50 years [6]. Some authors have been convinced of the evidence that a substantial proportion of foodborne disease is attributable to improper food preparation practices in consumers' homes [7,8]. One study estimated the proportion of food poisoning to be attributable to cross-contamination in the household kitchen to be 20% for *Salmonella*, 30% for *Campylobacter *and 40% for *E. coli *O157:H7. Based on this assessment the authors went onto argue that a targeted in home environmental disinfection campaign would be highly cost effective.

What is clear is that personal hygiene in the form of handwashing with soap or a substitute for soap is strongly protective of diarrhoeal disease risk in both developing and developed countries [9,10]. However, in a recent review, the point was made that evidence that home hygiene is protective against diarrhoeal disease comes largely from developing countries and or for situations out of the home [9,11]. There has been very few studies looking at the impact of in-home hygiene practices in developed countries. No formal systematic review has been conducted to assess the importance of household hygiene and issues surrounding food handling, preparation and storage practices and the development of diarrhoea in the developed world.

The objectives of this review were to examine if household hygiene in relation to food preparation, food handling and food storage practices are important contributors to the development of diarrhoea in developed countries.

## Methods

### Criteria for considering studies for this review

Both observational and intervention studies were included in this review. Those studies whose participants were households, children or adults from developed countries were included. Those types of exposure identified were around household, especially kitchen, hygiene and cleanliness or concerned food preparation and storage practices at home. The outcomes included were either self reported diarrhoea with no associated pathogen identified or cases of diarrhoea with a known enteric pathogen identified. Studies that looked only at hand washing or drinking water as factors were excluded. Studies conducted during outbreak investigations were also excluded.

### Search methods for identification of studies

The following databases were used with the search terms stated below:

1. MEDLINE (1966 to date)

2. EMBASE (1980 to date)

3. Web of Knowledge (-to date)

4. Cochrane Library register of Controlled Trials (CENTRAL), 2005

5. CINAHL (1982 to date)

The following search strategy was used:

1. Standardised search strategy

▪ cohort studies [MeSH Terms] OR controlled trials [MeSH Terms] OR case-control studies [MeSH Terms] OR ecological studies [MeSH Terms] OR odds ratio [Text Word] OR cross-sectional studies [MeSH Terms] OR case-control [Text Word] OR confidence interval [Text Word] OR relative risk [Text Word] OR observational studies [MeSH Terms]

This search was combined with the subject specific search stated below

2. Subject specific search strategy

▪ Diarrhoeal* [Text Word] OR Diarrhoea* [MeSH Terms] OR Gastroenteritis [Text Word] OR Campylobacter [Text Word] OR Salmonella [Text Word] OR Norovirus [Text Word] OR Escherichia Coli [Text Word]

*all spellings of diarrhoea were also tried with the American spelling; diarrhea

AND

▪ Food [Text Word] OR Food [MeSH Terms] AND preparation [MeSH Terms] OR preparation [Text Word] OR handling [Text Word] OR handling [MeSH Terms] OR storage [MeSH Terms] OR storage [Text Word] OR hygiene [Text Word] OR hygiene [MeSH Terms] OR hygiene [All fields] OR temperature [Text Word]

OR

▪ Household [MeSH Terms] OR household [Text Word] OR hygiene [Text Word] OR hygiene [MeSH Terms] OR hygiene [All Fields] OR cleanliness [MeSH Terms] OR cleanliness [Text Word] OR pets [Text Word] OR pets [MeSH Terms] OR dogs [Text Word] OR cats [Text Word] OR birds [Text Word] OR flies [Text Word] OR flies [MeSH Terms] OR insects [Text Word] OR insects [MeSH Terms]

### Selection of Studies

Each publication was independently assessed by two of us, with the third involved to resolve disagreements. The initial search identified 1378 studies. After independent screening of the titles, 378 studies were identified which was further narrowed to 48 studies following review of the abstracts and hard copies of these studies were obtained. In addition to the formal search, Web of Knowledge was searched to identify whether papers citing or cited by chosen papers were relevant, 14 studies were finally included in subsequent analyses. Of the 14 studies included in this systematic review, 11 were case-control studies [12-22], 2 cross-sectional surveys [23,24], and 1 RCT. (Table 1) [25].

**Table 1:** Results from reanalysis of UK IID GP case control study component.

		Control	Case	Unadjusted			Adjusted^a^		
				
				OR	95% CI	P	OR	95% CI	P
Owns pet27	No	681	754	1		0.0056			0.093
	Yes	1017	939	0.816	0.707 – 0.942		0.836	0.678 – 1.030	
Shares WC	No	1584	1565	1		0.260			
	Yes	91	103	1.185	0.882 – 1.593				
Shares kitchen	No	1581	1557	1		0.294			
	Yes	80	89	1.350	0.863 – 1.630				
Length of work surface	< 1 m	91	94	1.152	0.834 – 1.592		1.143	0.703 – 1.860	
	1 – 2 m	588	625	1.178	1.014 – 1.369		1.221	0.981 – 1.520	
	> 2 m	934	824	1		0.045	1		0.115
Owns fridge	No	79	89	1.153	0.841 – 1.581				
	Yes	1612	1608	1		0.377			
Owns freezer	No	75	111	1.479	1.094 – 1.999		1.219	0.761 – 1.951	
	Yes	1617	1586	1		0.011	1		0.411
Owns dishwasher	No	1172	1224	1.172	1.004 – 1.368		1.178	0.932 – 1.487	
	Yes	520	473	1		0.045	1		0.170
Soak nappies in kitchen	No	1657	1666	1		0.638			
	Yes	10	8	0.800	0.316 – 2.027				
Shop frequency	>1 per week	410	504	1		0.649			
	1 per week	1070	920	0.697	0.591 – 0.821				
	1 per 2 weeks	124	155	1.009	0.763 – 1.334				
	1 per month	65	92	1.112	0.778 – 1.590				
	Less often	6	4	0.521	0.145 – 1.869				
Checks use by date	Always	1312	1378	1		0.002	1		0.214
	Sometimes	321	229	0.696	0.575 – 0.843		0.792	0.596 – 1.051	
	Never	22	20	0.996	0.531 – 1.870		1.106	0.453 – 2.700	
Follows storage instructions	Always	1208	1282	1		3.3 × 10^-4^	1		0.020
	Sometimes	386	306	0.719	0.602 – 0.859		0.721	0.556 – 0.933	
	Never	30	23	0.940	0.597 – 1.482		0.803	0.383 – 1.682	
Check packaging for damage when shopping	Always	1363	1349	1		0.753			
	Sometimes	261	247	0.940	0.772 – 1.144				
	Never	15	17	1.197	0.589 – 2.433				
Pack frozen food together in cool container for transport	Always	364	430	1		0.049	1		0.312
	Sometimes	456	380	0.697	0.569 – 0.853		0.713	0.527 – 0.965	
	Never	791	751	0.804	0.671 – 0.963		1.060	0.812 – 1.382	
Check appearance of product when shopping	Always	1456	1420	1		0.572			
	Sometimes	180	181	1.034	0.825 – 1.295				
	Never	8	12	1.375	0.553 – 3.418				
Stores meat in fridge	On bottom shelf	964	868	1		0.0007	1		0.0009
	Anywhere else	740	837	1.269	1.106 – 1.457		1.419	1.155 – 1.742	
Use separate chopping board for raw and cooked meats	No	807	688	0.769	0.663 – 0.893		0.803	0.648 – 0.994	
	Yes	801	871	1		0.0006	1		0.044
Use separate chopping board for other raw and cooked foods	No	999	860	0.772	0.664 – 0.897		0.741	0.599 – 0.919	
	Yes	607	677	1		0.0007	1		0.006
Cleans chopping board between raw and cooked foods	No	52	55	1.064	0.714 – 1.584				
	Yes	1570	1496	1		0.761			
Uses same cloth for wiping all surfaces in kitchen	No	810	789	1		0.884			
	Yes	854	848	1.011	0.876 – 1.165				
Normally cools foods	In fridge	597	716	1		2.0 × 10^-7^	1		^b^
	In larder	77	83	0.938	0.657 – 1.339		0.651	0.388 – 1.093	
	On work surface	874	717	0.634	0.536 – 0.749		0.704	0.552 – 0.897	
Normally stores foods for eating later	In fridge	1549	492	1		0.334			
	In larder	16	15	1.097	0.500 – 2.405				
	On work surface	43	56	1.360	0.900 – 2.057				

^a^Adjusted for contact with ill person out of the home, visit overseas and lives in rented *vs *owner occupied home.

^b^Not calculated by software package.

### Data collection

A pre-piloted data extraction form (available from the authors) was used to extract and record data from the included studies. Key data that was extracted include subject characteristics, location, outcome (diarrhoea) and exposure (food storage, food handling, food preparation and hygiene practices), timing of exposure in relation to outcome, the obtained results and final conclusions.

In addition to the published studies, the primary data from the UK Intestinal Infectious Disease study was obtained from the UK Data-Archive [26]. From this data set the two files relating to Case Control study of people attending their General Practitioner with diarrhoea were extracted and merged. The UK Intestinal Infectious Disease study was a large prospective study of diarrhoeal disease. As part of this study a cohort of volunteers were followed up for six months in order to determine the population incidence of self-reported diarrhoea. This was compared with the incidence of diarrhoea as estimated from prospective surveillance of patients attending general practice and also from national surveillance data. The study was funded by the England Department of Health and has been described in detail elsewhere [3,16,27]. One element of this study was to identify risk factors in patients attending their family doctor with intestinal illness. Each case recruited into the study was matched by age and sex to another patient registered at the practice and both case and control asked to complete a lengthy questionnaire.

### Methodological Quality Assessment

Methodological quality assessment was based largely on whether or not the results were controlled for possible confounding variables. Low quality studies did not control for any confounding, medium quality studies controlled for age and gender, and high quality studies controlled for multiple confounding variables. If the exposure date was self-reported rather than observed then the quality score dropped one grade. The randomised controlled trial was not scored.

### Data Analysis

It was the intension to conduct meta-analyses of the data from published work. However, the heterogeneity of outcome measures, risk factors reported and indeed whether Odds Ratios were even presented prevented a meta analysis. All Odds Ratios were as given in the primary publication, though the reciprocals are presented for some such that any increased risk associated with poorer hygiene would give an OR of >1.0.

This GP case control data component of this study was analysed using StatsDirect version v 2.6.2 [28]. All hygiene related variables were analysed using conditional logistic regression for each predictor variables alone. For ordinal predictor variables the p value was calculated for trend as recommended by Agresti [29]. Further analyses were done on variables significant at p < 0.2 level and adjusted for the key potential confounding variables. The potentially confounding variables were contact with a person with diarrhoea outside of the home, visit overseas and living in rented accommodation which were derived from conditional logistic regression of non-hygiene and non-food consumption variables.

## Results

The results for the systematic review are shown in the Additional file 1. There was only one high quality study, nine medium and three low quality studies. The overwhelming consistency was that the vast majority of studies showed no association between markers of poor kitchen hygiene and the disease outcomes. Studies that showed any statistically significant result were one that reported a low hygiene score indicated increased risk of diarrhoea due to Rotavirus infection (OR 1.5 95% CI 1.1–2.1) [12]. This hygiene score reported cleanliness of cutting boards as an important independent factor, together with four other variables, e.g. duration of keeping eggs. However, rotavirus is an infection that is generally spread by person-to-person transmission rather than by food. The other studies with significant results were in low quality studies where there was inadequate attention to possible confounding variables and inadequate multivariable analyses. In one of these studies two of the three significant variables gave counter-intuitive significant associations with poor hygiene practices being associated with reduced risk. All other studies were not significant, although where ORs were given there was a general trend towards improved hygiene being associated with reduced illness, though confidence intervals were generally very wide.

There was only one randomised controlled trial [25]. The two arms of this study looked at using disinfectant or an identically packed sham product in kitchen cleaning. This RCT did not find any significant evidence that use of disinfectant in kitchen hygiene was protective against self reported diarrhoea. In the one case-control study that tested the association with the use of antibacterial agent and disease there was also no statistical association [13].

The results of the re-analysis of the UK IID study are shown in Table 1. There was no association in the adjusted analyses between self-reported diarrhoea and most variables indicative of increased risk of cross-contamination in the domestic kitchen (pet ownership, sharing a WC, sharing a kitchen, small length of work surface, owning a dishwasher, soaking nappies in kitchen, cleaning chopping board between raw and cooked foods and using the same cloth for wiping all surfaces in the kitchen). Factors associated with a lower risk of self-reported diarrhoea were not using separate chopping boards for raw and cooked meats (OR 0.803; 95%CI 0.648 – 0.994) or for other raw and cooked foods (0.741; 0.599 – 0.919). The factor associated with a higher risk was storing food anywhere in the fridge other than on the bottom shelf (1.419; 1.155 – 1.742).

Variables likely to be associated with bacterial regrowth in foods were also not associated with self-reported diarrhoea (owning a fridge or freezer, not storing foods for later in a fridge; packing frozen foods together for transport home) or were associated with a reduced risk of illness; only sometimes following food storage instructions (OR 0.721; 95%CI 0.556 – 0.933). On the other hand, normally cooling food on the work surface rather than a refrigerator was associated with a reduced risk (0.704; 0.552 – 0.897).

## Discussion

In both the systematic review and in the re-analysis of the UK IID study, little evidence was found to suggest that environmental hygiene practices in domestic kitchens reduce the morbidity rates of diarrhoeal disease. Indeed for several variables poor hygienic practices (using the same chopping board for raw and cooked meats and other raw and cooked foods) were negatively associated with illness. One potentially relevant risk factor linking cross contamination and increased disease risk was storing meat in a fridge other than on the bottom shelf. However, given the number of potential factors analysed it is difficult to draw any firm conclusions either way.

It should be noted that there was only one intervention study; the use of disinfectant in home cleaning compared to cleaning with a product with no disinfectant and that also did not find a significant effect. However, this study compared cleaning with and without a disinfectant product and did not compare cleaning with not cleaning.

One of the problems with this systematic review was that observational studies included used several different risk factors and used different endpoints. The risk factors for Salmonella, Campylobacter, Norovirus, Rotavirus and self-reported diarrhoea are likely to be different [30]. However, most episodes of diarrhoeal disease affecting people living in developed nations are probably due to viruses which are almost exclusively human only pathogens. Having said that, included in the review were papers that considered both Salmonella and Campylobacter infections, the commonest two bacterial causes of food poisoning. In none of these studies was kitchen hygiene factors associated with any increased risk.

One other problem when trying to identify risk factors from observational studies is that authors often do not list all potential risk factors included in the questionnaire if they are not statistically significant. This will lead to an under ascertainment of negative relative to positive studies. However, even if negative studies were missed this is unlikely to change the basic conclusions in this case.

In the re-analysis of the UK IID study, the most surprising finding was the counter-intuitive associations found. The issue of negative associations between potential risk factors and disease outcome has been discussed elsewhere [31]. In the context of this study, it is likely that negative associations were probably due to confounding in that the results were adjusted for only three, albeit highly significant, predictor variables. It is highly probable that if a full multivariate model was constructed all these associations would have become not significant. Indeed this was the case in one of the larger case-control studies in the review where generally protective associations in univariable analyses were not included in the final multivariable model [21]. One of the potential explanations for negative associations between risk factors and an infectious disease outcome is that frequent exposure to low dose exposure may protect against disease through development of increased immunity. There is some evidence for this hypothesis [32,33]. However, it is premature to suggest that this is the case in this situation.

When considering the questions that had been asked in the various studies to assess kitchen hygiene it became clear that the effectiveness of the questions varied depending on what their were attempting to quantify. It seems to us that those questions that attempted to assess the risk of cross-contamination would have been quite effective (e.g. use of the same chopping board for raw and cooked meet, use of the same cloth for all work surfaces). However, it seemed to us that there were few questions asked that would give a good estimate of the adequacy of thawing foods prior to cooking or the adequacy of cooking practices.

A further issue is the problem of recall bias in case control studies [34]. The particular problem is differential recall bias where cases are more likely than controls to recall an exposure thought by them to be the cause of their illness. However, such differential recall would more likely increase the apparent significance of any particular risk factor. On the other hand, it is possible that people with unhygienic domestic kitchens and hygiene practices may over estimate their personal hygiene. If this was the case then strength of any statistical association would be reduced.

If poor domestic kitchen domestic hygiene is not a risk factor for food poisoning, this raises the issue of what factors within the home are responsible for home transmission. Domestic acquired food poisoning can be due to purchase of ready to eat foods that are already contaminated. This is most obvious in outbreak settings [35-37]. There are many studies world-wide that have identified risk factors for sporadic food poisoning. The most common such risk factors have included consumption of raw or under cooked meat, poultry and eggs [38-44].

This review also raises the question of why the findings from this study appear to be so different from previous estimates [7,8]. In particular, the study that estimated the high proportion of disease preventable by in home disinfection is worth re-considering [8]. This study was funded by a disinfection manufacturer. The authors based their estimate on a mixture of expert opinion and the sole (low quality) study that found a strong association between kitchen hygiene and disease outcome [22]. In this context expert opinion was canvassed by asking a panel of experts to estimate the disease burden attributable to poor domestic kitchen hygiene. In their review of the literature, the authors did not include any of the other five (negative) and generally higher quality studies published before 2003 [13,15-17,19]. All of these studies would have been available to inform their estimates. The discrepancy between our findings and the outcome of an expert opinion exercise also raises concerns about the value of expert opinion in estimating disease attribution. The sources of error in expert opinion have been discussed elsewhere [45]. It seems to us that two sources of systematic bias are particularly worth mentioning. Firstly, people responsible for bringing together expert panels may consciously or sub-consciously choose people who they believe will lean towards one conclusion or another. Secondly, experts themselves may consciously or sub-consciously inflate the importance of their special area of interest by over-estimating the proportion of disease attributable to their area of expertise. There is a need to reassess how expert opinion is canvassed for risk assessment and, indeed, policy discussions. There is also an argument that expert opinion panels should be drawn up by people independently of the main researchers who may have vested interests and that panels are deliberately chosen to include experts known to have divergent opinions.

## Conclusion

In conclusion this review does not support the hypothesis that poor general environmental hygiene in the domestic kitchen is a risk factor for *Salmonella*, *Campylobacter *or self-reported diarrhoea. There is evidence that poor kitchen hygiene may be a risk factor for Enterohemorrhagic *E. coli *but this was a single low quality study with few cases and no adequate control for possible confounding [22]. However, all the data with one exception were based on observational studies and consequently no unequivocal conclusions can be drawn at this stage. It is doubtful that the impact of domestic kitchen hygiene will be firmly resolved based on cases control studies. We would argue that there is a need for properly conducted prospective cohort or randomised intervention studies to really investigate the contribution of particular domestic kitchen hygiene practices may or may not have on the risk of diarrhoeal disease.

## Competing interests

PRH has acted as an expert witness relating to food poisoning on several occasions for both claimants and defendants. All other authors have no competing interests to declare.

## Authors' contributions

All authors contributed equally to the study design and literature review. AS wrote the first draft. PRH did the statistical analyses. All authors read and approved the final manuscript.

## Pre-publication history

The pre-publication history for this paper can be accessed here:



## Supplementary Material

Additional file 1 Results obtained from systematic review of studies looking at food hygiene, preparation, storage and handling. This table lists all studies identified in the systematic review along with their key characteristics and findings. Click here for file
