# Supplementary material for: How effective is good domestic kitchen hygiene at reducing diarrhoeal disease in developed countries? A systematic review and reanalysis of the UK IID study
Source: BMC Public Health. 2008 Feb 22;8:71. doi: 10.1186/1471-2458-8-71 (PMC2266741; doi:10.1186/1471-2458-8-71)
Supplement: Additional file 1 — Results obtained from systematic review of studies looking at food hygiene, preparation, storage and handling. This table lists all studies identified in the systematic review along with their key characteristics and findings. [file 1471-2458-8-71-S1.doc]

Results obtained from systematic review of studies looking at food hygiene, preparation, storage and handling

| **Study** | **Quality** | **Population** | **Outcome** | **Cases** | **Controlled variables** | **Risk/Intervention** | **OR** | **95% CI** | **P** |
| --- | --- | --- | --- | --- | --- | --- | --- | --- | --- |
| **Case-control studies** | | | | | | | | | |
| 12 | Low | Netherlands, age-stratified sample, including all ages <1- >70 yrs, | Norovirus | 152 pairs | Age, Gender | Food handling hygiene score | 1.3 | 1.0 – 1.7 | - |
|  |  |  | Rotavirus | 54 pairs |  | Food handling hygiene score | 1.5 | 1.1 – 2.1 | - |
| 13 | Medium | United States, Louisiana residents >15yrs of age, | Salmonella | 115 case control pairs | Age, co-morbidities, education and ethnicity  Age, co-morbidities, education and ethnicity | Cutting surface wood vs plastic | 1.3 | 0.6 – 2.5 | - |
|  |  |  |  |  |  | Uses different surface for meat and non-meat items vs. the same | 0.9 | 0.5 – 1.5 | - |
|  |  |  |  |  |  | Cleaned chopping board each time b/w meat and non-meat items | 3.7 | 0.3 – 44.9 | - |
|  |  |  |  |  |  | Cleaned with soap/disinfectant/bleach vs water | 1.4 | 0.4 – 5.7 | - |
| 14 | Medium | Denmark | Campylobacter | 217 cases  236 control | Age, co-morbidities, education and ethnicity | Less thorough cleaning of cutting board | 1.47 | 0.66 - 3.33 | - |
|  |  |  |  |  |  | Less thorough cleaning of knives | 2.08 | 0.9 – 4.76 | - |
|  |  |  |  |  |  | Scalding cutting boards | 3.85 | 0.85 – 16.67 | - |
|  |  |  |  |  |  | Scalding sink | 1.22 | 0.15 – 10.0 | - |
| 15 | Medium | Australia, 0-35 month old children in Brisbane | Diarrhoea Campylobacter | 81 cases  144 controls | Age (within 3 months), gender, geographic location | Always washed utensils b/w raw and cooked foods vs. never | 2.00 | 0.14 – 27.59 | 0.86 |
|  |  |  |  |  |  | Room temperature vs. refrigerator/microwave | 1.25 | 0.63 – 2.47 | 0.56 |
| 16 | Medium | England | Campylobacter | 229 cases and controls | Age, Sex, GP practice, social class, education, employment, | Various measures of kitchen hygiene | Not stated | - | Not significant |
| 17 | Medium | Norway, , 1-66 yrs; mean age 24.8 yrs. | Campylobacter | 52 cases  103 controls | Age, Geographical location | Various domestic kitchen hygiene factors | Not stated | - | Not significant |
| 18 | Medium | South East Wales | Salmonella | 137 cases  99 controls | Age, sex | Salmonella isolated from dish clothes | 2.0 | 0.6 – 7.8 |  |
|  |  |  |  |  |  | Total bacterial and enterobacteriacea counts in dish clothes |  |  | Not significant |
| 19 | Medium | South East Wales | Salmonella | 137 cases  99 controls | season,  age of food handler, child <5  person >65 | Various measures of contact with raw poultry |  |  | Not significant |
|  |  |  |  |  |  | Various measures of potential for cross contamination including use of antibacterial cleaner |  |  | Not significant |
|  |  |  |  |  |  | Various measure of poor temperature control of stored foods |  |  | Not significant |
| 20 | Low | Australia | Self-reported diarrhoea | 687 cases  1134 controls | None | Frequently defrosting chicken in microwave | 2.5 | 1.5 – 4.0 | <0.001 |
|  |  |  |  |  |  | Where store food in refrigerator |  |  | Not significant |
|  |  |  |  |  |  | Handling of cutting boards including not washing or separating raw and cooked foods with exception of |  |  | Not significant |
|  |  |  |  |  |  | ‘always’ placing cooked chicken and meat where raw chicken/meat had been | 0.31 | 0.13 – 0.76 | 0.01 |
|  |  |  |  |  |  | likely cross-contamination by dishcloths |  |  | Not significant |
|  |  |  |  |  |  | How long or how often foods were left unrefrigerated with exception of |  |  | Not significant |
|  |  |  |  |  |  | ‘always’ leaving food outside the  refrigerator for more than 2 h | 0.47 | 0.23 – 0.96 | 0.04 |
|  |  |  |  |  |  | Frequency of hand washing after touching raw meet |  |  | Not significant |
| 21 | Medium | United States Food Net sites | Campylobacter | 1316 cases  1316 controls | Multivariable model | Various measures of kitchen hygiene |  |  | Not significant |
| 22 | Low | United States | *E. coli* O157:H7 | 23 cases  44 controls | None | Not washing hands after handling raw ground beef | 8.5 | 1.8 – 39.6 | 0.004 |
|  |  |  |  |  |  | Not washing surfaces with soap and water after contact with raw ground beef | 10.5 | 1.2 – 92.6 | 0.05 |
|  |  |  |  |  |  |  |  |  |  |
| **Cross-sectional surveys** | | | | | | | | | |
| 23 | Medium | Germany > 1 yr old living in private household | Self-reported diarrhoea | 510 subjects | Age, gender, co-morbidities, education | Various domestic kitchen hygiene factors | Not stated | - | Not significant |
| 24 | High | Hispanic community  New York  United States | Self-reported diarrhoea | 238 households  (1178 members) | Number of children < 6 yrs, number of subjects rating health as poor or fair, chronic co-morbidities, number of household members, number of people spending > 40 hrs outside the household per week, ethnicity, age, gender, location of birth | Cleanliness of cutting surface | Not stated | - | Not significant |
| **Randomised controlled trial** | | | | | | | | | |
| 25 | N/A | Hispanic community  New York  United States | Self-reported diarrhoea | 118 households in the control group (586 subjects) | Number of children < 6 yrs, number of subjects rating health as poor or fair, chronic co-morbidities, number of household members, number of people spending > 40 hrs outside the household per week, ethnicity, age, gender, location of birth | Randomised control trial of use of two different products for kitchen cleaning, one with and one without disinfectant, | 1.11 | 0.67 – 1.85 | >0.2 |
